# Structure of an ancestral ADP-dependent kinase with fructose-6P reveals key residues for binding, catalysis, and ligand-induced conformational changes

**DOI:** 10.1074/jbc.RA120.015376

**Published:** 2020-12-24

**Authors:** Sebastián M. Muñoz, Victor Castro-Fernandez, Victoria Guixé

**Affiliations:** Laboratorio de Bioquímica y Biología Molecular, Departamento de Biología, Facultad de Ciencias, Universidad de Chile, Santiago, Chile

**Keywords:** ADP-dependent kinase, fructose-6-phosphate, ancestral enzyme, enzyme structure, X-ray crystallography, phosphofructokinase, glucokinase, archaea, substrate specificity, F6P, fructose-6-phosphate, GK, glucokinases, PFK, phosphofructokinases

## Abstract

ADP-dependent kinases were first described in archaea, although their presence has also been reported in bacteria and eukaryotes (human and mouse). This enzyme family comprises three substrate specificities; specific phosphofructokinases (ADP-PFKs), specific glucokinases (ADP-GKs), and bifunctional enzymes (ADP-PFK/GK). Although many structures are available for members of this family, none exhibits fructose-6-phosphate (F6P) at the active site. Using an ancestral enzyme, we obtain the first structure of an ADP-dependent kinase (AncMsPFK) with F6P at its active site. Key residues for sugar binding and catalysis were identified by alanine scanning, D36 being a critical residue for F6P binding and catalysis. However, this residue hinders glucose binding because its mutation to alanine converts the AncMsPFK enzyme into a specific ADP-GK. Residue K179 is critical for F6P binding, while residues N181 and R212 are also important for this sugar binding, but to a lesser extent. This structure also provides evidence for the requirement of both substrates (sugar and nucleotide) to accomplish the conformational change leading to a closed conformation. This suggests that AncMsPFK mainly populates two states (open and closed) during the catalytic cycle, as reported for specific ADP-PFK. This situation differs from that described for specific ADP-GK enzymes, where each substrate independently causes a sequential domain closure, resulting in three conformational states (open, semiclosed, and closed).

Glycolysis is one of the most important cellular processes to obtain ATP in all living organisms, being universally conserved. Nonetheless, in some archaea from the phylum Euryarchaeota, a modified version of this pathway has been described, where glucose and fructose-6-phosphate (F6P) are phosphorylated by homologous enzymes that use ADP instead of ATP as phosphoryl donor (1). These ADP-dependent sugar kinases can be specific for glucose (ADP-glucokinase [ADP-GK]), fructose-6P (ADP-phosphofructokinase [ADP-PFK]), or bifunctional able to use both substrates (ADP-PFK/GK). Sequence analysis, phylogenetic closeness, and the presence of conserved motifs associate to bifunctionality, leading to propose that the ADP-PFK from the order *Methanosarcinales* would be bifunctional enzymes. However, the kinetic characterization of two enzymes from this order shows that these enzymes have a marked preference for F6P, unlike the enzymes from *Methanococcales* (2).

The ADP-dependent sugar kinases belong to the ribokinase superfamily and their members display a highly two-domain conserved architecture, a large domain with a Rossmann-like fold and a small α/β domain that acts a lid of the active site (3). To date, many crystal structures of ADP-GK from hyperthermophilic archaea belonging to the order *Thermococcales* have been determined at high resolution. ADP-GK structures from *Thermococcus litoralis* (*Tl*GK, Protein Data Bank [PDB] codes: 1GC5, 4B8R, and 4B8S) (3, 4), *Pyrococcus furiosus* (*Pfu*GK, PDB code: 1UA4) (5), and *Pyrococcus horikoshii* (*Ph*GK, PDB codes: 1L2L and 5O0J) (6, 7) in apo form or complexed with ligands are available. Moreover, the structure of a truncated N-terminal variant of the mouse ADP-GK has been reported (*Mm*GK, PDB codes: 5CCF and 5CK7) (8). For ADP-PFK enzymes, the only structure determined corresponds to the enzyme from *P. horikoshii* in the apo form and in complex with AMP (*Ph*PFK, PDB codes: 1U2X and 3DRW) (9). Recently, the structure of a bifunctional ADP-PFK/GK from *Methanocaldococcus jannaschii* (*Mj*PFK/GK, PDB code: 5OD2) was also reported. This was the first structure of an ADP-PFK/GK enzyme belonging to the order *Methanococcales* (10), in addition to structures of ancestral enzymes of this family (PDB codes: 5K27, 5KKG, and 6C8Z) (11, 12). A comparison of the structures of ADP-dependent GKs in the apo form and in complex with ligands from different species indicates that both substrates (ADP and glucose) induce a conformational change, which has been suggested to be critical to the enzyme function. Moreover, a comparison of ADP-GK from *T. litoralis* bound to different ligands reveals three conformational states with sequential changes induced by both ligands that correlate with its kinetic mechanism. This ligand-induced conformational change brings the two domains closer through a hinge formed by flexible loops located between the large and small domains (1).

Interestingly, when the structure of the ADP-PFK from *P. horikoshii* in its apo form was superimposed with that obtained in complex with AMP, no such conformational change was observed. Moreover, because most of the structures of this family correspond to GK enzymes, plenty of information is available about residues involved in glucose binding and in catalysis. Unfortunately, despite the efforts performed by several groups, no crystal structure of an ADP-dependent kinase with F6P at its active site is available to date. These efforts include the specific ADP-PFK from *P. horikoshii* (*Ph*PFK), where cocrystallization and soaking trials in the presence of F6P, fructose-1,6-bisP, along with ADP or nonhydrolyzable analogs of nucleotide were performed (9). Also, trials with enzymes such as ADP-PFK/GK from *Methanococcoides burtonii* (*Mb*PFK/GK) (2), *Methanosarcina mazei* (*Mmaz*PFK/GK), and *Methanohalobium evestigatum* (*Meve*PFK/GK) were unsuccessful (12). The absence of such a structure has precluded studies that identify key residues for F6P binding and catalysis. To address this issue, we focus our efforts on obtaining a crystal structure of an ADP-PFK complexed with F6P. We choose the last common ancestor of the order *Methanosarcinales* because the protein can be obtained soluble with a high yield and its structure complexed with Mg-ADP (PDB code: 6C8Z) has been recently reported (12). Sequence analysis shows the presence of the conserved motif associates with bifunctionality, and kinetic characterization demonstrated that this ancestral enzyme could employ F6P or glucose as substrates, being a valuable model to address key residues responsible for sugar binding. Nonetheless, the kinetic parameters for both substrates suggest that glucose phosphorylation can be considered a promiscuous activity. Moreover, because the AncMsPFK structure in the presence of Mg-ADP is available, we can evaluate the impact of ligand binding on the conformational changes relevant to catalysis. Here, we report the crystallographic structure of an ancestral ADP-PFK in ternary complex with F6P and Mg-ADP_βS_, being the first structure of an enzyme from the ADP-dependent family with F6P at its active site. This structure represents a closed conformation compared with the ADP-PFK structures reported to date.

## Results

### Overall structure

Structures of other ancestral proteins from this enzyme family, such as the AncMT-PFK/GK and AncMsPFK (PDB codes: 5K27 and 6C8Z) are reported, but only in the presence of nucleotide (AMP and Mg-ADP, respectively) (11, 12). The structure of the AncMsPFK in complex with F6P-Mg-ADP_βS_ was solved by molecular replacement and refined at 3.12 Å resolution to R*work*/R*free* values of 0.24/0.27. Statistics for data collection and refinement are shown in Table 1. The AncMsPFK crystal contained one molecule per asymmetric unit with 460 of the expected 490 residues, and the unit cell corresponds to P 2_1_2_1_2_1._ In the final model, 97.8% of the residues were in favored regions of the Ramachandran plot and none were in the disallowed region.

**Table 1:** Crystallographic data and refinement statistics for AncMsPFK-F6P

Data collection	
X-ray source	LNLS MX2
Detector	PILATUS 2M
Wavelength (Å)	1.459
Resolution range (Å)	46.54–3.12
Space group	P 2_1_ 2_1_ 2_1_
Unitary cell (Å, °)	a = 67.57 b = 79.59 c = 108.57 α = β = γ = 90
Total reflections/unique reflections	135,177/10,902
Multiplicity	12.4 (12.1)
Completeness	98.33 (99.53)
Mean I/σ (I)	16.5 (3.02)
R-merge	0.1507 (0.841)
R-pim	0.04486 (0.2532)
CC1/2	0.996 (0.9)
Refinement	
R-*work*/R-*free*	0.2436/0.2772
Number of atoms	
Protein	3,540
Ligands	44
Waters	5
*B*-factors	
Protein	85.90
Ligands	77.48
Waters	66.90
RMS deviations	
Body lengths (Å)	0.005
Bond angles (°)	1.06
Ramachandran plot	
Favored (%)	97.81
Outside (%)	0.00
Rotamer outliers (%)	0.54
PDB code	6XIO

PDB, Protein Data Bank.

Statistics for the highest resolution shell are shown in parentheses.

**Figure 1:**
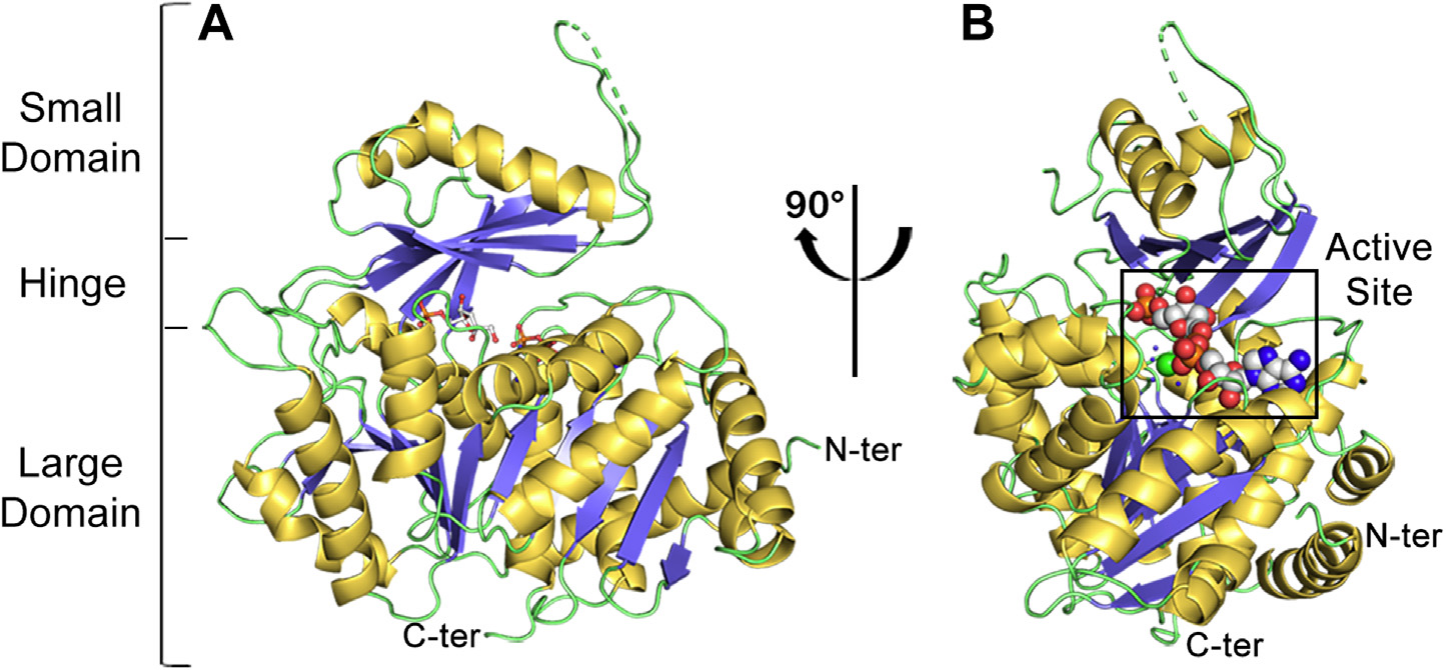
**Crystallographic structure of AncMsPFK in ternary complex with F6P-Mg-ADP**_**βS**_**.***A*, cartoon representation colored according to secondary structure: α-helices are colored in *yellow*, β-strands in *blue*, and loops in *green*. F6P and ADP_βS_ molecules are shown as *sticks*. *B*, the view of AncMsPFK active site. F6P, ADP_βS_, and Mg^2+^ ion are shown as *spheres*. F6P, fructose-6-phosphate.

The structure of AncMsPFK displays the classic fold described previously for ADP-dependent kinases, a large domain containing an α/β/α Rossmann-like fold with eleven stranded β-sheets surrounded by 14 α-helices, and a small domain formed by seven stranded β-sheets and four α-helices (3). However, 28 residues from the small domain cannot be solved in this structure; 24 of them belong to helices α2 and α3 and 4 from a β-turn located between β-sheets β9 and β10 (Fig. 1*A*). The lack of electronic density in these areas of the small domain in the closed conformation of AncMsPFK could result from the intrinsic flexibility of these regions. Also, for the small domain, a high average B-factor of 121 Å^2^ was refined compared with the 77.5 Å^2^ of the large domain (Fig. S1). In the same way, the average B-factor of the small domain in the closed structure of AncMsPFK is higher than values obtained for other ADP-PFK open conformations structures, such as Mg-ADP bound AncMsPFK or AMP-bound *Ph*PFK. This value is even higher than the one reported for other closed conformation structures, such as the *Mj*PFK/GK (Table S1).

**Figure 2:**
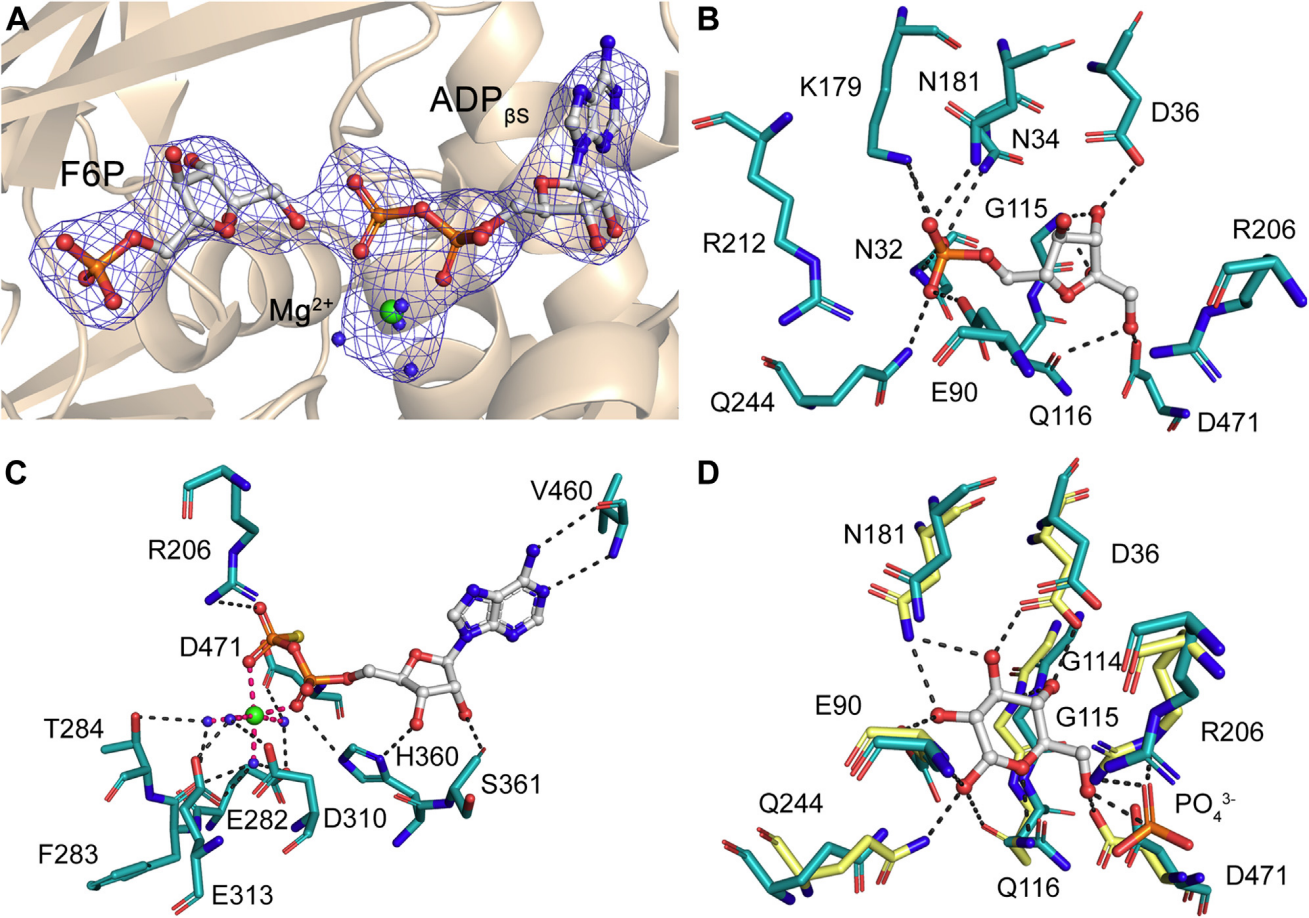
**Active site of AncMsPFK.***A*, close-up view on the AncMsPFK active site. F6P and ADP_βS_ molecules are shown as *sticks*, Mg^2+^ ion and water molecules are shown as *green* and *blue spheres*, respectively. *Blue mesh* indicates a Polder omit map contoured at 3 σ. *B*, polar interactions of F6P. *C*, polar interactions of Mg-ADP_βS_. Metal interactions are shown as *pink dotted lines*. *D*, superposition of AncMsPFK and *Mj*PFK/GK (PDB code: 5OD2) sugar-binding sites. Glucose molecule and PO_4_^3−^ ion of *Mj*PFK/GK are shown as *sticks*. Residues from AncMsPFK and *Mj*PFK/GK are shown as *cyan* and *yellow sticks*, respectively. Residues are labeled according to the AncMsPFK numeration. *Black dotted lines* indicate polar contacts calculated with PyMOL. F6P, fructose-6-phosphate.

The active site is located in a cleft between both domains (Fig. 1*B*), connected by a region of flexible loops that act as a hinge during the ligand-induced conformational change. The electronic density at the active site of the AncMsPFK structure allows us to assign both substrates, F6P and the Mg-ADP_βS_ complex, being the first structure of an ADP-dependent kinase with F6P at its active site (Fig. 2*A*). Analysis of residues involved in sugar binding shows that F6P is mainly stabilized by hydrogen bond interactions (Fig. 2*B*). The ribose ring interacts with D36, G115, Q116, and D471 residues. The strictly conserved D471 residue belongs to the GXGD motif and corresponds to the catalytic base that activates the hydroxyl group of the sugar to be phosphorylated, for the nucleophilic attack toward the ADP (1). The phosphate group makes contacts with N32, N34, E90, N181, K179, and Q244. Analysis of residues around F6P within a 5 Å radius identified M113, G114, I119, I183, I208, S210, S211, R212, M245, V467, and G468 (Fig. S2). In the theoretical docking model of ADP-PFK from *P. horikoshii* bounded with AMP, the R212 residue establish a direct interaction with the phosphate group of F6P (9), which is not observed in the AncMsPFK structure (Fig. 2*B*).

On the other hand, the Mg-ADP_βS_ complex is located in a shallow pocket in the large domain establishing hydrogen bonds and hydrophobic interactions with protein residues. The adenine moiety is surrounded by the side chains of residues L362, A395, V459, P469, and I469 and makes two hydrogen bonds with the main chain of V460. The ribose ring established these interactions with the H360 and S361 residues, whereas the β-phosphate interacts with R206 (Fig. 2*C*). Atoms of the lateral chain of residue R206 showed a high B-factor and a poor electronic density. However, this residue is highly conserved in the ADP-dependent family and has been pointed out to interact with the phosphoryl group of ADP, stabilizing the transfer reaction intermediary. These interactions are conserved among ADP-dependent sugar kinases and are also present in the AncMsPFK structure in the presence of Mg-ADP (PDB code: 6C8Z) (12). Despite the relatively low resolution of the structure, four water molecules around the Mg^2+^ ion were located, assuming its classical octahedral coordination geometry, which is in agreement with the electronic density of that region. Thus, the lateral chain of residues E282, T284, D310, E313, and D471 and the main chain of F283 interact indirectly with the Mg^2+^ ion through water molecules. Residue E282 belongs to the conserved HXE motif, whereas residues E313 and D310 belong to the DXXE motif, both of which are critical for Mg-ADP binding and ternary complex formation in the ADP-dependent sugar kinases family (13).

### Assessment of key residues involved in sugar binding

To gain insights into the key residues responsible for sugar binding, we search for a structure similar to AncMsPFK with glucose at its active site. Using the web Dali server (14), we found that the bifunctional ADP-PFK/GK from *M. jannaschii* (*Mj*PFK/GK, PDB code: 5OD2) was the most similar structure, with a 46% of identity, an RMSD for Cα of 1.9 Å over 425 residues, and a Z factor of 48. This structure was obtained in complex with glucose, the 5-iodotubercidine inhibitor, inorganic phosphate, and a Mg^2+^ ion at the active site. A structural alignment of both structures reveals that most of the residues that interact with glucose (D36, E90, G114, G115, Q116, N181, Q244, and D471) are present in the same position in the AncMsPFK structure except residues E90, Q116, and Q244. These residues appear to be located far away from the sugar in the ancestral enzyme, probably because of the presence of the phosphate group of F6P (Fig. 2*D*). Also, residue R206 of AncMsPFK is present in a different position compared with the glucose bound structure. This can be attributed to the inorganic phosphate emulating the β-phosphate of the ADP, which interacts with this arginine residue.

Structural alignment of the ancestral enzyme with the specific GK from *T. litoralis* bound to glucose and AMP (*Tl*GK, PDB code: 4B8S) shows that N181 of AncMsPFK is the only residue not conserved among those interacting with glucose (Fig. S3). In *Tl*GK, this residue is replaced by histidine in agreement with the substrate-specificity motifs described for this family (2).

**Figure 3:**
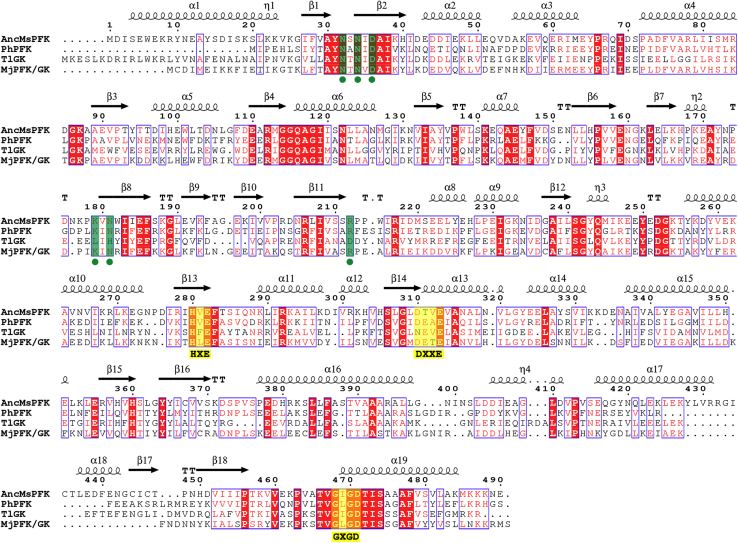
**Multiple sequence alignment of ADP-dependent kinases.***Ph*, *Pyrococcus horikoshii* (PFK-specific) (UniProt code: O59355); *Tl*, *Thermococcus litoralis* (GK-specific) (UniProt code: Q7M537), and *Mj*, *Methanocaldococcus jannaschii* (bifunctional) (UniProt code: Q58999). Conserved motifs are highlighted in *yellow*. Hinge residues responsible for substrate binding are marked with a *green dot*. The similarity-coloring scheme is based on the blosum62 matrix, and secondary structure elements were retrieved from AncMsPFK structure (PDB code: 6C8Z). The figure was generated with ESPript 3.0. GK, glucokinase; PDB, Protein Data Bank; PFK, phosphofructokinase.

To evaluate the role of active-site residues, we mutated residues N32, D36, E90, K179, N181, R206, and R212 by alanine. The mutants were kinetically characterized to assess their role in F6P, as well as in glucose binding and catalysis. Among the residues selected, N32, D36, and R206 are highly conserved in the whole family of ADP-dependent kinases, whereas residues K179, N181, and R212 are only conserved in specific ADP-PFK and bifunctional enzymes. On the other hand, the E90 residue is only conserved in specific ADP-GKs and bifunctional enzymes (Fig. 3).

Table 2 shows the kinetic parameters for both sugar substrates, glucose, and F6P for the WT enzyme and the selected mutants. For the PFK activity, D36A and K179A mutants display the most significant K_M_ increase for F6P, around 1,000-fold compared with the WT enzyme, whereas for the N181A and R212A mutants, this increase is approximately 200-fold. Regarding the catalytic constant (k_cat_), the most significant change was observed in the D36A mutant, where this value decreases 400-fold compared with the WT enzyme. Owing to the decrease in the catalytic constant and the high glucose concentrations needed to determine the kinetic parameters for the GK activity, a high error in the estimation of the K_M_ values of some mutants is observed. The highest increase in the K_M_ for glucose was observed in the N181A mutant (around 6-fold), whereas for the N32A and E90A mutants, this value increases 4- and 2-fold, respectively. The most significant diminution in the k_cat_ (180-fold) is obtained when the E90 residue was mutated to alanine. Unexpectedly, the D36A mutant displays the most remarkable diminution in the K_M_ for glucose (approximately 200-fold) and only a slight decrease in the k_cat_, which provokes an increment in the catalytic efficiency of about two orders of magnitude. Although this residue interacts with the C3 and C4 hydroxyl groups of glucose, this result suggests that the aspartic residue would hinder glucose binding. On the other hand, the most dramatic effect was observed in the R206A mutant, where no PFK or GK activity was detected in the assay conditions used for the other mutants. Efforts to detect PFK activity using discontinuous assays and reaction time of 18 h allow us to estimate a PFK activity of about 2 × 10^−6^ U/mg. This value represents a diminution of 600 thousand–fold respect to the WT enzyme, which precludes the estimation of the kinetic parameters for this mutant.

**Table 2:** Kinetic parameters for WT and AncMsPFK mutants

Enzyme	F6P				Glucose			
	K_M_ mM	k_cat_ s^−1^	K_i_ mM	k_cat_/k_M_ M^−1^s^−1^	K_M_ mM	k_cat_ s^−1^	K_i_ mM	k_cat_/k_M_ M^−1^s^−1^
WT	0.014 ± 1.5 × 10^−3^	1.2 ± 0.03		8 × 10^5^	131 ± 19	0.09 ± 5 × 10^−3^		0.69
N32A	0.18 ± 0.04	0.03 ± 2 × 10^−3^	58 ± 15	165	545 ± 184	0.006 ± 1 × 10^−3^		0.011
D36A^a^	14 ± 1	0.003 ± 1 × 10^−4^		0.2	0.65 ± 0.08	0.045 ± 2 × 10^−3^	63 ± 9	69
E90A^b^	0.09 ± 9.6 × 10^−3^	0.015 ± 4 × 10^−4^	65 ± 13	163	307 ± 69	5 × 10^−4^ ± 5 × 10^−5^		1.6 × 10^−3^
K179A	13 ± 2	0.04 ± 1.5 × 10^−3^		3	113 ± 17	0.04 ± 2 × 10^−3^		0.35
N181A	2.8 ± 0.3	0.4 ± 0.02	77 ± 12	139	744 ± 89	0.02 ± 2 × 10^−3^		0.03
R212A	2.9 ± 0.3	1.9 ± 0.04		645	201 ± 32	0.14 ± 0.01		0.70

F6P, fructose-6-phosphate; GK, glucokinases.

The standard error was calculated from two independent measurements.

a The GK activity of the D36A mutant was calculated from three independent measurements.

b For the E90A mutant, both activities were calculated from three independent measurements.

### Ligand-induced conformational change: structural comparison between ADP-dependent GK and PFK enzymes

**Figure 4:**
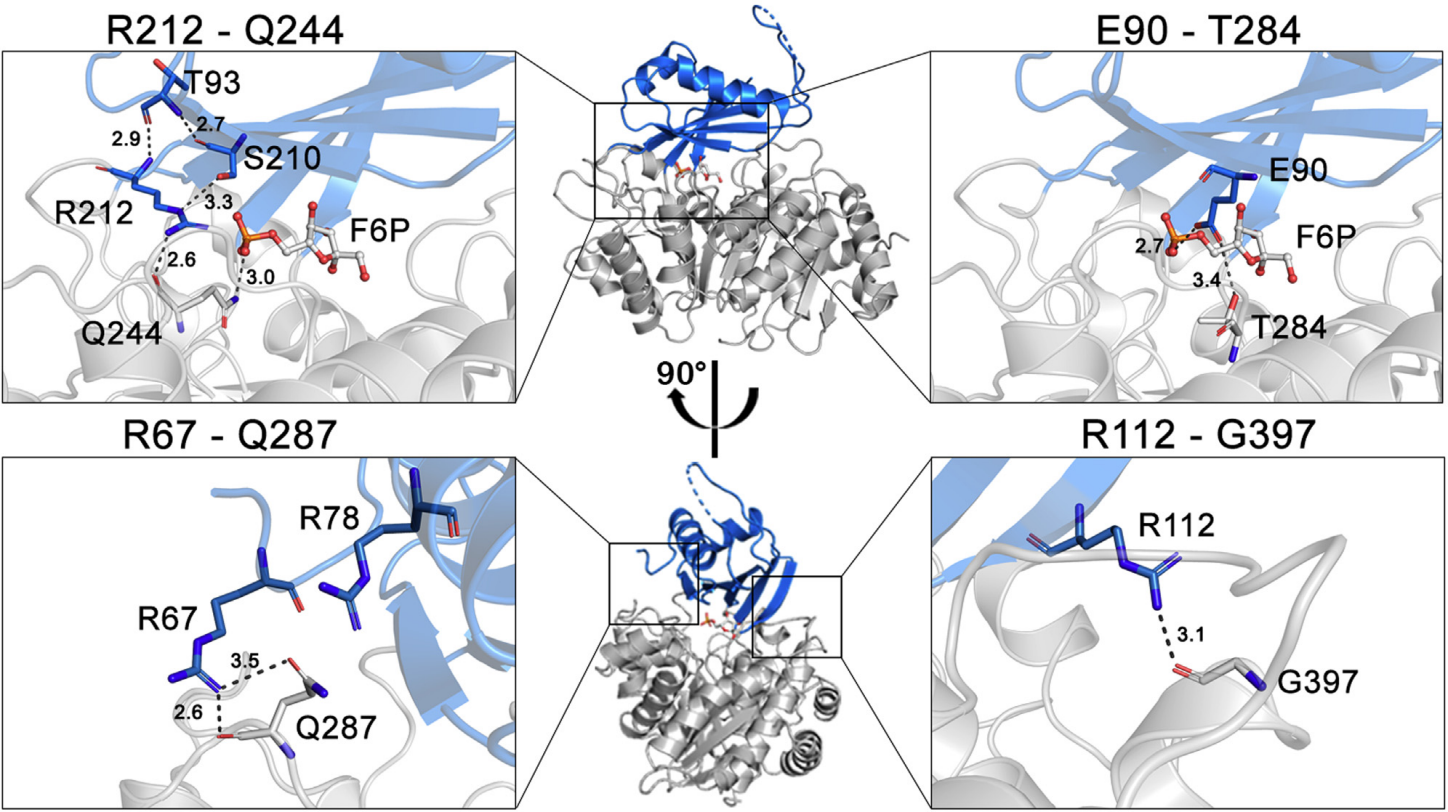
**Interactions between the small and large domains that stabilizes the closed conformation of AncMsPFK.** AncMsPFK structure is shown in a *cartoon* representation; the large domain is colored in *gray*, whereas the small domain is colored in *blue*. Interactions are displayed as a close-up view from AncMsPFK. Residues from interaction groups and F6P are shown as *sticks*. *Black dotted lines* indicate polar contacts, and distances (Å) were calculated with PyMOL. F6P, fructose-6-phosphate.

Analysis of residues involved in the stabilization of the close conformation of AncMsPFK reveals several interactions between the large and the small domains, governed by hydrogen bonds. These polar interactions include residues located close to F6P (R212-Q244 and E90-T284), and others such as arginine residues, far away from this sugar (R67-Q287 and R112-G397). Moreover, residue R78 is within an appropriate distance to interact with Q287 (Fig. 4). These interactions are present only in the ternary complex structure, being absent in the ADP-bound structure, supporting its stabilizing role in the closed state of the protein.

**Figure 5:**
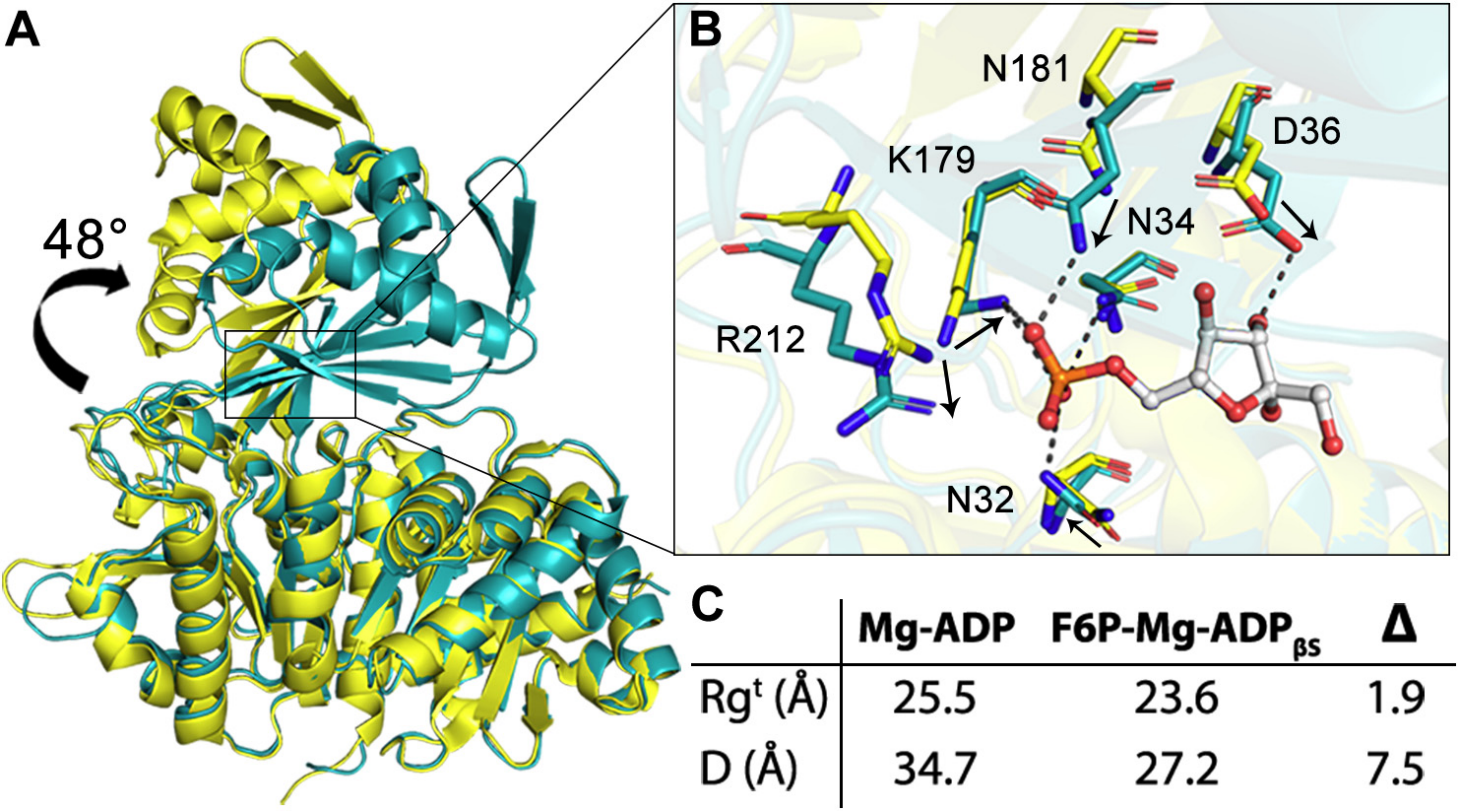
**Ligand-induced conformational change of AncMsPFK.***A*, structural alignment of AncMsPFK structures in the open (Mg-ADP) and closed (F6P-Mg-ADP_βS_) conformations, colored in *yellow* and *cyan*, respectively. The structural alignment was performed with PyMOL, whereas the rotation angle was calculated using DynDom web server. *B*, the close-up view of the hinge residues that participate in F6P binding. *Black arrows* indicate residue displacement orientation upon F6P binding. *C*, AncMsPFK theoretical radius of gyration (Rg^t^) calculated with CRYSOL web server and distance (D) between the center of mass of the small and large domains in the open (Mg-ADP, PDB code: 6C8Z) and closed (F6P-Mg-ADP_βS_) conformations. F6P, fructose-6-phosphate.

Further analyses of ligand-induced conformational changes were performed after modeling the missing secondary structure elements of the AncMsPFK small domain, using the small domain of the AncMsPFK structure bound to Mg-ADP as a template (PDB code: 6C8Z). A comparison of the open conformation of AncMsPFK (Mg-ADP) and the complete model in the closed conformation (F6P-Mg-ADP_βS_) shows an RMSD of 5.4 Å for the Cα positions. Analysis of the theoretical radius of gyration (Rg^t^) using the web CRYSOL server (15) indicates that the model in the presence of F6P-Mg-ADP_βS_ has a domain closure of 1.9 Å when compared with the structure in the presence of Mg-ADP. This difference is even more dramatic if the distance between the large and small domain center of mass was compared; in the closed conformation, both domains are 7.5 Å closer than in the structure with Mg-ADP at the active site (Fig. 5*A*).

A more detailed inspection of the conformational change using the DynDom web server (16) shows a small-domain movement of 48° as a consequence of F6P binding (Fig. 5*B*). In this movement, 22 residues act as a hinge (Table S2), where residues N32, N34, D36, K179, N181, and R212, responsible for sugar substrate binding, can be found (Fig. 5*C*). Half of them are located in β-sheets regions (N34, D36, and N181) and the other ones in loops (N32, K179, and R212) (Fig. S4).

## Discussion

Ancestral sequence reconstruction has been traditionally used to obtain information regarding the evolutionary history of a protein family (17). However, recently, it has also been proposed as a powerful tool for protein engineering, drug discovery, and for other applications, such as the development of biocatalysts (18). Moreover, by this approach, we were able to obtain a crystal structure of an ADP-dependent kinase in the presence of F6P, which could not be possible previously with extant proteins.

Because this family comprises specific enzymes for glucose, F6P or bifunctional (glucose and F6P), the lack of a structure in the presence of F6P has precluded obtaining empirical evidence for key residues responsible for sugar substrate binding and specificity. Then, taking advantage of an ancestral enzyme previously reconstructed, we report the first structure of an ADP-dependent sugar kinase in a ternary complex with F6P and Mg-ADP_βS_. This structure corresponds to a closed conformation that enables us to identify residues responsible for sugar binding and catalysis.

Previous kinetic characterization of the ancestral protein showed it was able to catalyze the phosphorylation of either glucose or F6P being the K_M_ for F6P similar to the one reported for other current enzymes from the order *Methanosarcinales* (*Mmaz*PFK/GK and *Meve*PFK/GK). Nonetheless, the K_M_ for glucose is around 10-fold higher than these enzymes (12) and 82-fold higher when compared with the *M. jannaschii* ADP-PFK/GK from the order *Methanococcales* (10). This result suggests that, like bifunctional enzymes from *Methanosarcinales*, the ancestral enzyme behaves like an ADP-PFK with promiscuous activity with glucose, despite the presence of the bifunctional motif reported for this family (2).

The AncMsPFK structure determination with F6P allows the identification of key residues for sugar binding. Among the residues selected for alanine scanning, N32 and D36 seem to be involved in the binding of both sugars; D36 interacts directly with F6P and glucose and N32 with F6P only. Superposition of the ancestral structure with *Mj*PFK/GK shows no direct interaction of the N32 residue with glucose, even when it is close to it. In the ADP-GK from *P. furiosus*, the interaction of this residue with glucose is mediated by water molecules (5).

Nonetheless, the most dramatic effect in the increase in the K_M_ for F6P, as well as in the diminution of k_cat,_ was observed in the D36A mutant. These results agree with those reported by Currie *et al.* (9), who mutated the corresponding residue in the ADP-PFK from *P. horikoshii*, sustaining the role of this residue in F6P binding and catalysis. Unexpectedly, this mutant shows a huge diminution in the K_M_ value for glucose and only a slight diminution in the k_cat_ value, which results in a very large increase in the catalytic efficiency for glucose. Moreover, the K_M_ value for glucose is similar to the one reported for specific GK, suck as the *Tl*GK enzyme (K_M_ = 0.22 mM) (4). Although this residue appears to be involved in the stabilization of the glucose ring in the available GK structures, our results suggest that this residue would impair glucose binding in the bifunctional AncMsPFK because its mutation by alanine makes the enzyme specific for glucose.

Sequence analysis of the ADP-dependent enzymes reveals that D36A mutation is absent in all the enzymes from the ADP-dependent family reported to date, even in specific GK enzymes. This suggests a restriction of this evolutive pathway to generate specific GK enzymes. In the scenario that this mutation occurs during evolution, it would correspond to a positive selection mechanism for glucose and, at the same time, to a negative one for F6P. It would be interesting to assess if this evolutive route toward glucose specificity is restricted in this family of enzymes because directed evolution experiments (19) and natural evolution of enzyme–substrate specificity (17) support a negative selection mechanism as the easiest way to generate specificity.

Residues K179 and R212 are conserved in specific ADP-PFK and bifunctional enzymes. In the AncMsPFK structure, K179 interacts directly with the phosphate group of F6P and R212 is too far away to establish any interaction with the sugar. These results differ from those proposed based on the docking of F6P performed in the theoretical model of the ADP-PFK from *P. horikoshii* bounded to AMP (9). Arginine in the *Ph*PFK model interacts directly with F6P, whereas the lysine is too far away to establish hydrogen bonds with the sugar. However, mutation of K179 by alanine in the AncMsPFK produces a noticeable increase in the K_M_ for F6P, whereas mutation of R212 produces a lower effect. Interestingly, the same result was obtained for alanine mutant of these arginine and lysine residues in *Ph*PFK (9).

Among residues involved in glucose binding, E90 is conserved in specific ADP-GK and bifunctional enzymes. A critical role in glucose binding has been described for this residue in another bifunctional ancestral enzyme from the orders *Thermococcales* and *Methanococcales* (11). Mutation of this residue in the AncMsPFK provokes a considerable diminution in the k_cat_ of the GK reaction and only a discrete increase in the K_M_, which demonstrated that the role of this residue is mainly in catalysis, probably by glucose orientation in the phosphoryl transfer reaction, more than in glucose binding.

Mutation of the R206 residue produces an inactive enzyme either for PFK or GK activities. Efforts to detect PFK activity in a discontinuous assay allow us to measure a diminution of approximately 600-thousand-fold in the specific activity. Also, mutation of the corresponding residue by alanine or lysine in ADP-GK from *T. litoralis* (*Tl*GK) produces an enzyme lacking catalytic activity (3). These results support the critical role of this residue in catalysis with both substrates.

Among the interactions that stabilize the close conformation of AncMsPFK, three of them involve arginine residues that form hydrogen bonds with residues from the small and the large domains. Another hydrogen-bond interaction is established between E90 with F6P, and probably has a role in the orientation of this sugar, considering the 80-fold diminution in the k_cat_ when it is mutated by alanine.

A comparison of the structure of AncMsPFK in the open conformation (in the presence of Mg-ADP) to the one obtained in the presence of F6P and MgADP_βS_ (closed conformation) shows that the sugar substrate induced a domain closure of 48°, where 22 hinge residues take part. The *in silico* analysis performed with the bifunctional *Mj*PFK/GK, modeled in the open and closed conformations, also shows a domain closure of 47° where 13 hinge residues participate (20). Some hinge residues in both enzymes are involved in sugar specificity, like N32, N34, D36, K179, N181, and R212, for the ancestral enzyme. In the ADP-bound structure, residues D36, N181, and R212 are too far from the active site, and substrate interaction can be established only after ligand-induced domain closure. On the other hand, the orientation of the lateral chain of residues N32, N34, and K179 changes upon sugar binding, which allows the interaction of these residues with F6P, inducing the closed conformation (Fig. 5*C*). Besides, residue E90 interacts with the sugar substrate only in the closed conformation, contributing to its stabilization.

Binding of F6P to the active site provokes a diminution of 1.9 Å in the theoretical radius of gyration and a difference of 7.5 Å in the distance between the center of mass of the large and small domains of AncMsPFK. However, in the specific ADP-GK from *T. litoralis*, smaller changes between the semi-closed (ADP, PDB code: 1GC5) and the closed conformations are observed, being 0.5 Å for the radius of gyration and 1.7 Å for the center of mass (4). This suggests a more significant sugar-induced conformational change in PFK than in GK enzymes.

**Figure 6:**
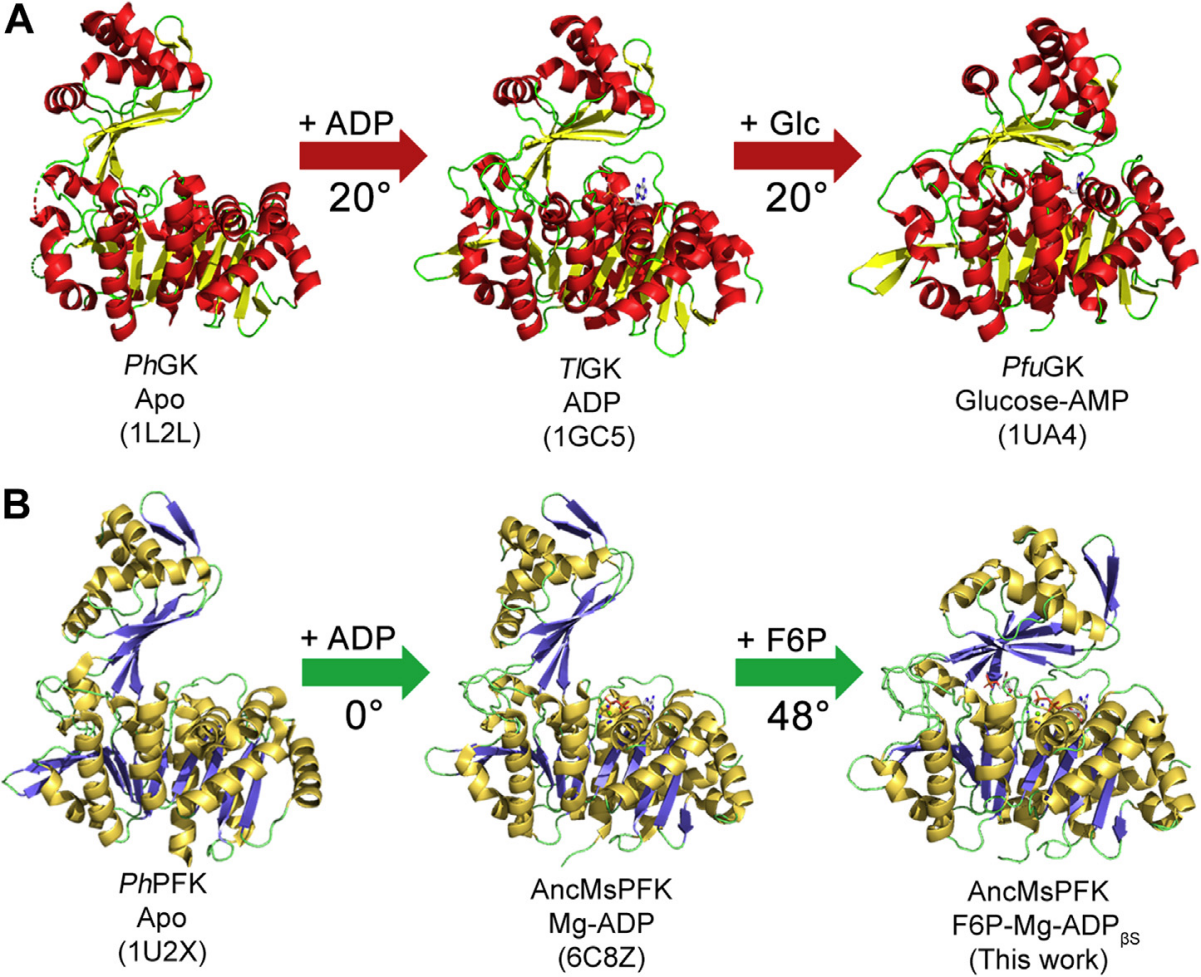
**Differential ligand-induced conformational changes between ADP-GKs and ADP-PFKs.***A*, conformational changes induced by ADP and glucose (Glc) in ADP-GK structures. *B*, conformational change induced by ADP and F6P in ADP-PFK structures. For each structure, its PDB code and the rotation angle of the small domain is shown. F6P, fructose-6-phosphate; GK, glucokinase; PDB, Protein Data Bank; PFK, phosphofructokinase.

Interestingly, a comparison of specific ADP-GK enzymes in different ligation states demonstrated a sequential event of conformational change along with substrates binding from the apo form (open conformation) to a semiclosed conformation (ADP bound) and finally to a closed conformation (ternary complex with ADP and glucose bound). A comparison of the apo *Ph*GK (PDB code: 1L2L) corresponds to the most open conformation reported, with the *Tl*GK structure in the presence of ADP (PDB code: 1GC5) showing a 20° movement of the small domain. Moreover, if this last structure is compared with the *Pfu*GK enzyme in ternary complex with glucose and AMP (PDB code: 1UA4), an additional displacement of the small domain of 20° is observed (Fig. 6*A*). Also, sequential conformational changes involving three conformational states are reported for the *Tl*GK enzyme, although for this case, changes of small magnitude are observed (4).

However, based on the structure reported here, this sequence of ligand-induced conformational changes would be absent in ADP-PFK enzymes. For example, no conformational change is observed between the structures of *Ph*PFK in the apo form (PDB code: 1U2X) and complex with AMP (PDB code: 3DRW). In this case, the authors hypothesized that the binding of both the nucleotide and the sugar would be necessary to achieve the closed conformation or perhaps is the β-phosphate of the ADP molecule responsible for the conformational change (9). Also, a comparison of the apo form of *Ph*PFK with the structure of the AncMsPFK complexed with ADP (12) reveals no conformational change, being both structures in an open conformation. Nonetheless, a comparison of the ancestral enzyme in its open form with the structure obtained in the presence of F6P-Mg-ADP_βS_ shows a remarkable domain closure of 48°. This support that for ADP-PFK enzymes, binding of both substrates is required to achieve the closed conformation (Fig. 6*B*). Moreover, the available crystallographic data suggest that for ADP-PFK, two conformations (open and closed) could be accessible, whereas ADP-GK enzymes would populate three conformational states, open, semiclosed, and closed. The exception to this scenario is the recently reported structures of a truncated ADP-GK from mouse in the apo form (PDB code: 5CCF) and in the presence of AMP (PDB code: 5CK7), where no conformational change is detected. In this case, the absence of the small domain displacement could be because the AMP structure was obtained by soaking the crystals of the apo form (8). Although small angle X-ray scattering) or cryo-electron microscopy methodologies would be very suitable for addressing the conformational states of ADP-PFK enzymes, both involved major experimental challenges. For the first one, according to the theoretical Rg values, the structure will undergo a domain closure of 2 Å, which would need very good quality small angle X-ray scattering data. On the other hand, cryo-electron microscopy experiments with low-molecular-weight proteins are difficult because although the size limits are continuously challenged, only a few structures below <100 kDa are reported (21).

Finally, three conserved residues from the hinge in specific ADP-PFK would be relevant for the conformational change (K179, N181, and R212 in the AncMsPFK) because these residues also participate in F6P binding. Then, in these enzymes, the sugar substrate-binging would be necessary to accommodate the hinge residues and generate the interactions required to accomplish the conformational change that leads to the closed conformation.

## Experimental procedures

### Protein purification

The sequence of the last common ancestor of ADP-PFKs of *Methanosarcinales* (AncMsPFK) was expressed and purified as described previously (12). Briefly, the ancestral sequence was inferred using the Hierarchical Bayesian method (22), and the gene sequence was codon-optimized for expression in *Escherichia coli* and synthesized by GENSCRIPT (Piscataway, NJ). The gene was cloned into the pET-15 (NOVAGEN) expression vector, which includes a polyhistidine-tag at the N-terminus and ampicillin resistance.

AncMsPFK was cloned in BL21 *E. coli* cells, and protein expression was induced at *A*_0.6_ with 1-mM IPTG overnight at 37 °C. Cells were centrifuged (8000*g* for 15 min at 4 °C), suspended in lysis buffer (25-mM Tris HCl, pH 7.8, 20-mM imidazole, 1 M NaCl, and 5-mM MgCl_2_), disrupted by sonication (12 pulses of 20 s, with 40% amplitude and 1-min intervals), and centrifuged at 15,000*g* for 20 min at 4 °C. The supernatant was heated at 80 °C for 20 min, centrifugated at 15,000*g* for 20 min at 4 °C, and loaded onto a high-performance Ni^+2^-Sepharose 5-ml column (GE Healthcare), equilibrated with buffer A (25-mM Tris HCl, pH 7.8, 20-mM imidazole, 500-mM NaCl, and 5-mM MgCl_2_). The protein was eluted with a linear gradient of 80- to 200-mM imidazole in the same buffer. The fractions with enzyme activity were pooled and dialyzed against a protein storage buffer (25-mM Tris HCl, pH 7.8, 300-mM NaCl, 5-mM MgCl_2_, and 50% glycerol) and stored at −80 °C. The yield was ∼60 mg of pure protein per liter of culture. The AncMsPFK mutants were purified using the same protocol as for WT enzyme.

### Site-directed mutagenesis

The AncMsPFK mutants N32A, D36A, E90A, K179A, N181A, R206A, and R212A were constructed using the QuikChange II site-directed mutagenesis kit (Agilent Technologies) according to the manufacturer’s protocol using the pET-15_AncMsPFK expression vector as the template. Oligonucleotides for site-directed mutagenesis are listed in Table S3.

### Enzyme activity measurements

PFK and GK ADP-dependent activities of AncMsPFK WT and mutants were determined as previously reported (23). The ADP-PFK activity was measured by following the oxidation of NADH at 340 nm in a coupled assay with the following auxiliary enzymes: a-glycerol-3-phosphate dehydrogenase (5 units), triosephosphate isomerase (50 units), and aldolase (1.3 units) all from rabbit muscle (Sigma-Aldrich), along with 100-mM Pipes, pH 6.5, 0.2-mM NADH, 2-mM MgCl_2_, and 1-mM ADP. The GK-ADP activity was determined by following the reduction of NAD^+^ at 340 nm in a coupled assay with 0.5 units of G6PDH from *Leuconostoc mesenteroides*, which was heterologously expressed in *E. coli* and purified in our laboratory. For both assays, the reaction volume was 0.2 ml. For mutants exhibiting a huge reduction in its ADP-GK activity (N32A and E90A), and therefore with very low initial rates, only 0.05 units of G6PDH from *L. mesenteroides* were added to the assay mixture, to reduce the promiscuous activity of the auxiliary enzyme at high glucose concentrations. The assay also contained 100-mM Hepes, pH 7.8, 0.5-mM NAD^+^, 2-mM MgCl_2_, and 1-mM ADP. All enzyme activity determinations were performed using a microplate reader (Sinergy 2, Biotek) at 25 °C and the specific activity (U/mg) was calculated from the initial velocity data. One unit of enzyme activity (U) was defined as the amount of enzyme which catalyzes the formation of 1 μmol of product per minute. Kinetic parameters for PFK and GK-ADP activities were determined at a fixed concentration of its cosubstrate (Mg-ADP) and varying concentration of the sugar substrate (F6P or glucose). The Michaelis–Menten or substrate inhibition equations were fitted to the data by nonlinear regression.

### Protein crystallization

Purified AncMsPFK with a polyhistidine tag was screened for crystallization conditions with commercial kits using an ARI Gryphon crystallization robot (Art Robbins Instruments LLC) by the sitting-drop vapor-diffusion method. Preliminary crystals of AncMsPFK in complex with F6P–MgADP_βS_ were obtained using the PEGRx reagent kit (Hampton Research Corp), with subsequent rounds of optimization. Good-quality crystals were obtained at 5 mg/ml of AncMsPFK using 1 μl of protein (6-mM F6P, 2.4-mM ADP_βS_, 7.4-mM MgCl_2_, 25-mM Hepes, pH 7.8, 200-mM NaCl, and 1-mM 2-mercaptoethanol) and 1 μl of reservoir solution containing 8.4% w/V PEG 20000, 100-mM sodium citrate, pH 5.2, and 200-mM MgCl_2_. Crystals appeared after 72 h at 18 °C. AncMsPFK crystals were soaked into cryoprotective solution Parabar 10312 (Hampton Research) and flash-frozen in liquid nitrogen.

### X-ray diffraction, structure determination, and refinement

X-ray diffraction data were collected at 100K on the MX2 beamline at the Brazilian Synchrotron Light Laboratory (LNLS Campinas-SP) using a PILATUS 2M detector (Dectris Ltd) and an X-ray wavelength of 1.459 Å. The data were indexed, integrated, and scaled with XDS (24) and merged using Aimless from the CCP4 package (25). The phases were solved by molecular replacement with Molrep (26), using the structure of the ancestral AncMsPFK in complex with Mg-ADP (PDB code: 6C8Z) (12), which was split into the small and large domains as search models. The structure was refined using Phenix (27) and COOT (28). The F6P, ADP_βS_, magnesium ion, and waters molecules were placed using COOT and refined with restraints generated in ReadySet/eLBOW in Phenix. Validation of the structure was performed in the web server MolProbity (29). Full data collection and refinement statistics are summarized in Table 2. Figures were performed with PyMol and electronic density in Figure 2*A* was obtained from a Polder omit map (30).

### Multiple sequence alignment

The AncMsPFK sequence was aligned against the previous alignment reported for the ADP-dependent kinase protein family (2). The structure of the ADP-PFK from *P. horikoshii*, ADP-GK from *T. litoralis*, and ADP-PFK/GK from *M. jannaschii* (PDB codes: 3DRW, 1GC5, and 5OD2) was structurally aligned using STAMP. The target sequences were aligned with ClustalW using the structural alignment as a profile. Complementarily, this alignment was corrected by visual inspection in MultiSeq from VMD. The figure was generated with ESPript 3.0 (31).

### Protein homology model

To model the missing secondary structure elements in the small domain of AncMsPFK structure, a homology model was constructed using MODELLER, v 9.15 (32). Fifty models were generated using as template the experimentally determined structure and the small domain of a previously determined structure of AncMsPFK (PDB code: 6C8Z) (12). Models were evaluated based on their discrete optimized protein energy potential, and the quality of the resulting models was assessed in the web server MolProbity (29).

## Data availability

The structure presented in this article has been deposited in the Protein Data Bank (PDB) with the following code: 6XIO. All remaining data are contained within the article.

## Conflicts of interest

The authors declare that they have no conflicts of interest with the contents of this article.
