# Supplementary material for: Structure of an ancestral ADP-dependent kinase with fructose-6P reveals key residues for binding, catalysis, and ligand-induced conformational changes
Source: J Biol Chem. 2020 Dec 24;296:100219. doi: 10.1074/jbc.RA120.015376 (PMC7948494; doi:10.1074/jbc.RA120.015376)
Supplement: Supplementary Figures and Tables [file mmc1.pdf]

Structure of an ancestral ADP-dependent kinase with fructose-6P reveals key residues for binding, catalysis, and ligand-induced conformational changes

Sebastian M. Muñoz[1], Victor Castro-Fernandez[1*], Victoria Guixé[1*].

[1]Laboratorio de Bioquímica y Biología Molecular, Departamento de Biología, Facultad de Ciencias, Universidad de Chile, Santiago, Chile.

*Corresponding authors: Victor Castro-Fernandez and Victoria Guixé

E-mails: vcasfe@uchile.cl, vguixe@uchile.cl.

Supporting Information

Figure S1. B-factor representation of AncMsPFK structure.

Figure S2. First shell contacts in fructose-6P binding.

Figure S3. Comparison of AncMsPFK and *Tl*GK sugar-binding sites.

Figure S4. Hinge residues of AncMsPFK.

Table S1. B-factor for ADP-PFK structures.

Table S2. Hinge residues of AncMsPFK.

Table S3. Oligonucleotides for site-directed mutagenesis.

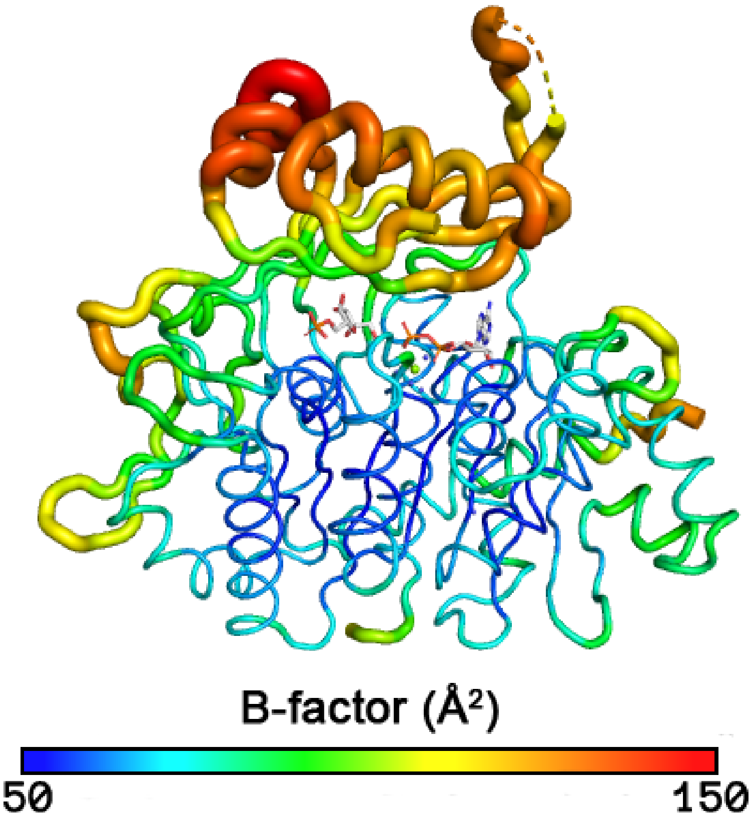

Figure S1. B-factor representation of AncMsPFK structure. The structure of AncMsPFK is represented as a cartoon colored according to the B-factor values of different protein regions . A color scale indicates the minimum (blue) and maximum (red) B-factor values. Fructose-6P and $ADP_{\beta S}$ molecules are shown as sticks. The figure was generated with PyMOL.

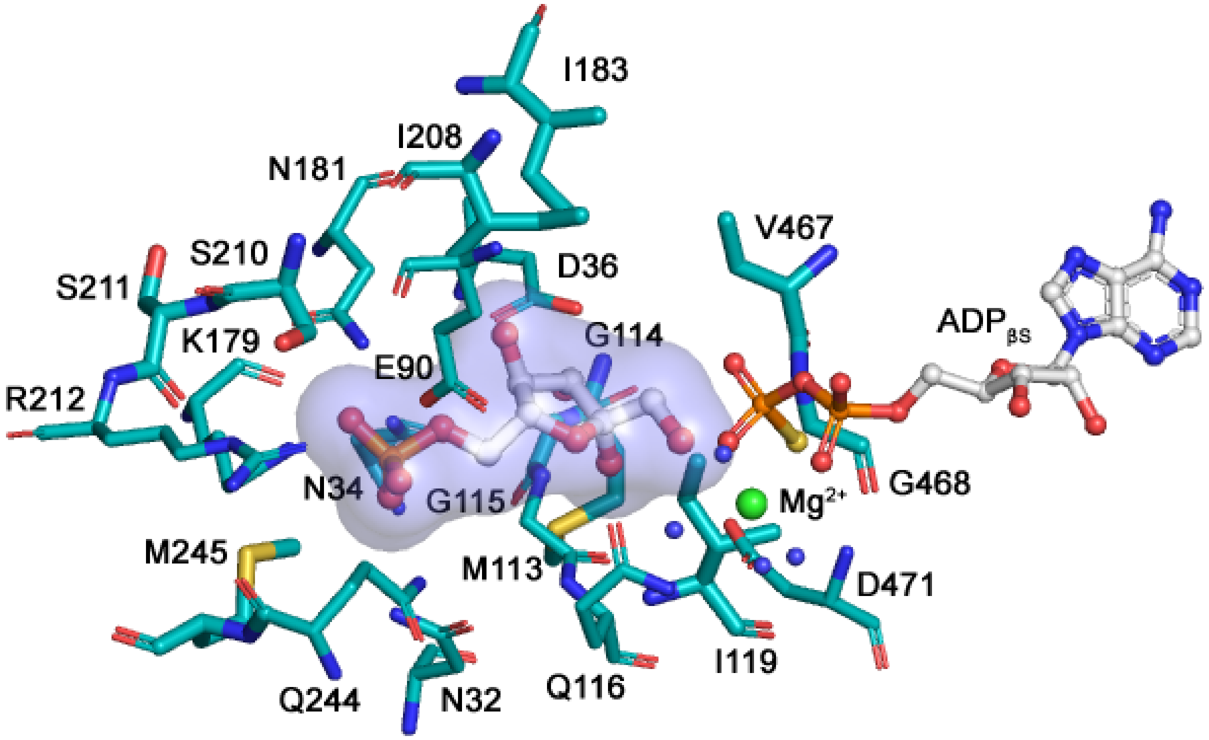

Figure S2. First shell contacts in fructose-6P binding. Residues around 5 Å distance from Fructose-6P were calculated in PyMOL. Fructose-6P molecule is shown as sticks. The blue surface around F6P correspond to the surface area displayed by PyMOL. The Mg^{2+} ion is shown as a green sphere and coordination waters are shown as blue spheres.

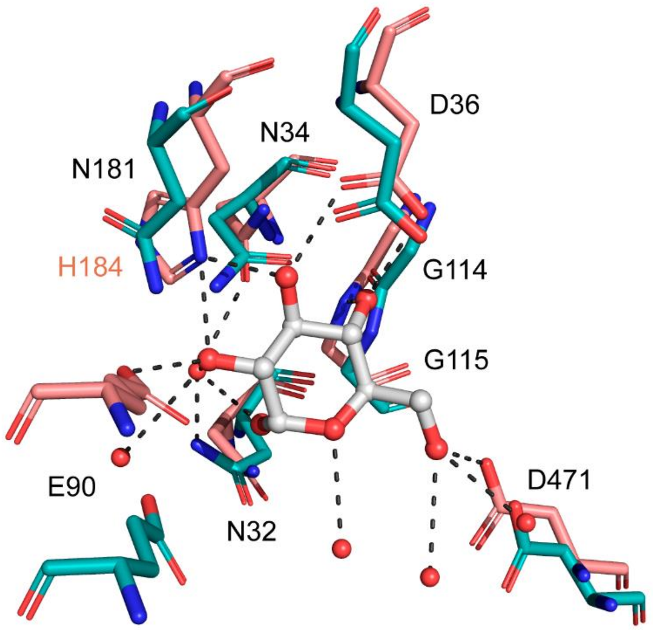

Figure S3. Comparison of AncMsPFK and *Tl*GK sugar-binding sites. Structural superposition of AncMsPFK and *Tl*GK (PDB code: 4B8S) sugar-binding sites. The glucose molecule is shown as sticks. Residues from AncMsPFK and *Tl*GK are shown as cyan and pink sticks, respectively. Residues are labeled according to the AncMsPFK numeration (except for H184 labeled according *Tl*GK numeration). Black dotted lines indicate polar contacts calculated with PyMOL.

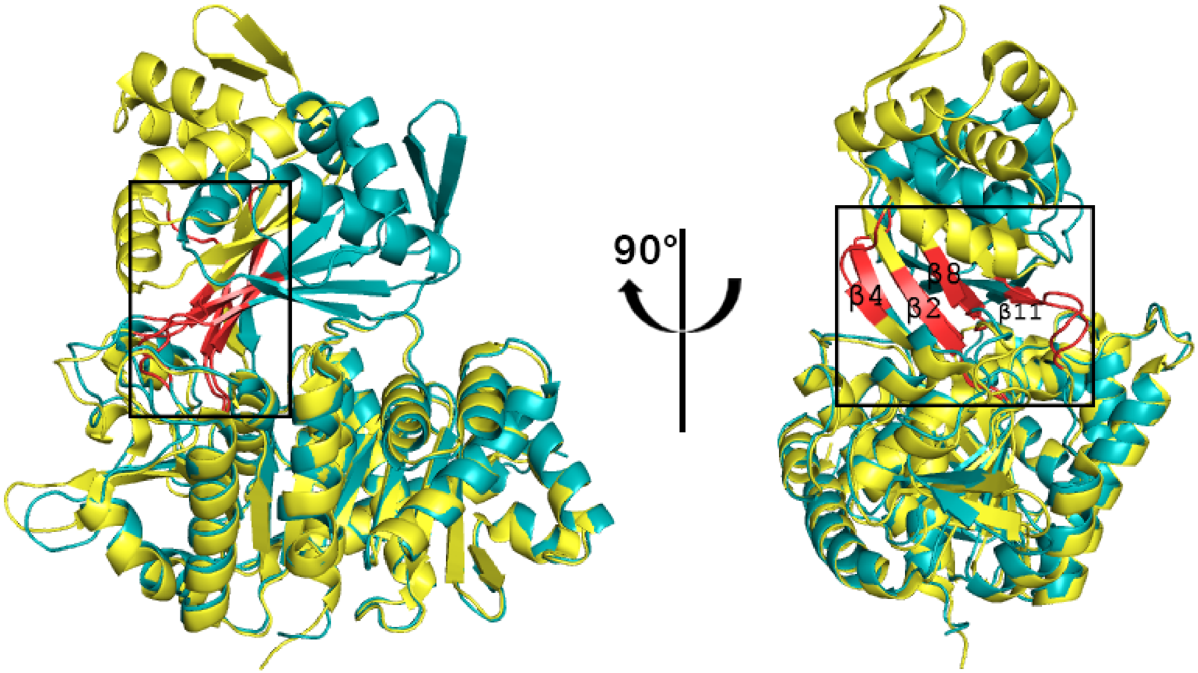

Figure S4. Hinge residues of AncMsPFK. Structural alignment of AncMsPFK structures in the open (Mg-ADP) and closed (F6P-Mg-ADP$_{\beta S}$) conformations are shown in yellow and cyan, respectively. Secondary structure elements and loops of the hinge are shown in red.

Table S1. B-factor for ADP-PFK structures.

Enzyme	PDB ID	Conformation	Ligands	Resolution (Å)	B-factor (Å²)	Mean B-factor (Å²)	
AncMsPFK	6XIO	Close	F6P	3.12	85	Large Domain	77.5
			Mg-$ADP_{\beta S}$			Small Domain	121.0
						Delta	43.5
AncMsPFK	6C8Z	Open	Mg-ADP	2.86	68	Large Domain	65.7
						Small Domain	78.3
						Delta	12.6
*Ph*PFK	3DRW	Open	AMP	1.90	29	Large Domain	27.3
						Small Domain	32.2
						Delta	4.9
*Mj*PFK/GK	5OD2	Close	Glucose 5IOD*	1.98	25	Large Domain	28.0
						Small Domain	29.5
						Delta	1.5

** 5IOD corresponds to inhibitor 5-iodotubercidine.*

Table S2. Hinge residues of AncMsPFK.

Hinge residue	Secondary structure
Asn32	Loop β1/β2
Ser33	Loop β1/β2
Asn34	β-sheet 2
Ile35	β-sheet 2
Asp36	β-sheet 2
Ala37	β-sheet 2
Gly107	Loop α5/β4
Phe108	Loop α5/β4
Asp109	Loop α5/β4
Glu110	β-sheet 4
Ala111	β-sheet 4
Pro178	Loop β-turn/β8
Lys179	Loop β-turn/β8
Val180	β-sheet 8
Asn181	β-sheet 8
Trp182	β-sheet 8
Ser211	β-sheet 11
Arg212	Loop β11/β-turn
Pro213	Loop β11/B-turn
Pro214	β-turn
Trp215	β-turn
Ile216	Loop β-turn/α8

**Residues highlighted in gray are also responsible for substrate specificity*

Table S3. Oligonucleotides for site-directed mutagenesis.

	Sequences
**N32A**	Fw: 5’-ggtatctttgttgcgtatgccagcaatatcgatgccattaaac-3’<br>Rv: 5’-gtttaatggcatcgatattgctggcatacgcaacaaagatacc-3’
**D36A**	Fw: 5’gttgcgtataacagcaatatcgcggccattaaacatatcgacgaag-3’<br>Rv: 5’-cttcgtcgatatgtttaatggccgcgatattgctgttatacgcaac-3’
**E90A**	Fw: 5’-ggtaaagcggccgcagttccgacctacac-3’<br>Rv: 5’-gtgtaggtcggaactgcggccgctttacc-3’
**K179A**	Fw: 5’-catataacccggataataaaccggcagtcaactggatcatcgaattcag-3’<br>Rv: 5’-ctgaattcgatgatccagttgactgccggtttattatccgggttatatg-3’
**N181A**	Fw: 5’-cccggataataaaccgaaagtcgcctggatcatcgaattcagtaaag-3’<br>Rv: 5’-ctttactgaattcgatgatccaggcgactttcggtttattatccggg-3’
**R206A**	Fw: 5’-gtgccgcgtgataatgccctgatcgtgagctc-3’<br>Rv: 5’-gagctcacgatcagggcattatcacgcggcac-3’
**R212A**	Fw: 5’-ctgatcgtgagctctgctccgccgtggattc-3’<br>Rv: 5’-gaatccacggcggagcagagctcacgatcag-3’
**AncMsPFK gene**	atggatatttctgaatgggaaaaacgttataacgaagcgtacagtgacattagtaaatccctgaagaaagtgaaaggtatctttgttg<br>cgtataacagcaatatcgatgccattaaacatatcgacgaagatgacattgaaaaactgctggaacaggtcgatgccaaagaagt<br>gcaagaacgtattatggaatatccgcgccagatcgattccccggcggactttgtggcccgtctgattatctcaatgcgcgatggtaa<br>agcggccgaagttccgacctacaccacggacattcacgaatggctgacggataacctgggcttcgacgaagcacgcatgggcg<br>gtcaggctggtattatctctaacctgctggcgaatatgggcattaaaaatgtcatcgcctatgtgccgtggctgtcgaaagaacaag<br>cggaatacttcgtggatagcgaaaacctgctgcatccggtggttgaaaatggcaaactggaactgaaacacccgaaagaagcat<br>ataacccggataataaaccgaaagtcaactggatcatcgaattcagtaaaggtctggaagttaaattcgctggcgaaaaaattgtcg<br>tgccgcgtgataatcgcctgatcgtgagctctcgtccgccgtggattcgtattgacatgtctgaagaactgtacgaacatctgccgg<br>aaattggcaaaaatatcgatggtgccattctgagtggctaccagatgatcaaagaagaatacgaagatggtaaaacctataaagac<br>tacgtggaaaaagcagtcaacgtgattaaacgtctgaaagaaggcaatccggatatccgcattcacgttgaatttaccagcatcca<br>aaacaaactgatccgtaaagcaattctgaaagatatcgttcgcaaacatgtccactcactgggtctggacacggttgaagtcgcga<br>acgccctgaatgtgctgggctatgaagaactggcatactcggttattaagaaagatgaaaacgcaatcgttgctctgtatgaaggtg<br>ctgtcattctgctgcatgaactgaaactggaacgtgtgcatgttcactcactgggctattacatctgcgttgtctcaaaagattcgcct<br>gtgagcccggaagaccaccgtaaatctctgctgttcgcaagtaccgttgcagctgcgcgcgctctgctgggtaacattaattcgct<br>ggatgacatcgaagccggtctggatgtccctgtgagcgaacagggctataaccaactggaaaaactggaaaaatacctggtgcg<br>tcgcggtatttgtaccctggaagattttgaaaacggctgcatctgtaccccgaatcatgacgtgattatcattccgacgaaagtggtt<br>gaaaaaccggtggcgaccgttggcattggtgatacgatctccgccgcagctttcgtttcagtcctggccaaaatgaaaaagaaaa<br>acgaa
